# Assembly of 913 microbial genomes from metagenomic sequencing of the cow rumen

**DOI:** 10.1038/s41467-018-03317-6

**Published:** 2018-02-28

**Authors:** Robert D. Stewart, Marc D. Auffret, Amanda Warr, Andrew H. Wiser, Maximilian O. Press, Kyle W. Langford, Ivan Liachko, Timothy J. Snelling, Richard J. Dewhurst, Alan W. Walker, Rainer Roehe, Mick Watson

**Affiliations:** 1 The Roslin Institute and R(D)SVS, University of Edinburgh, Easter Bush, EH25 9RG UK; 2 Scotland’s Rural College, Edinburgh, EH25 9RG UK; 3 Phase Genomics, 4000 Mason Road, Seattle, WA 98195 USA; 4 The Rowett Institute, University of Aberdeen, Aberdeen, AB25 2ZD UK

## Abstract

The cow rumen is adapted for the breakdown of plant material into energy and nutrients, a task largely performed by enzymes encoded by the rumen microbiome. Here we present 913 draft bacterial and archaeal genomes assembled from over 800 Gb of rumen metagenomic sequence data derived from 43 Scottish cattle, using both metagenomic binning and Hi-C-based proximity-guided assembly. Most of these genomes represent previously unsequenced strains and species. The draft genomes contain over 69,000 proteins predicted to be involved in carbohydrate metabolism, over 90% of which do not have a good match in public databases. Inclusion of the 913 genomes presented here improves metagenomic read classification by sevenfold against our own data, and by fivefold against other publicly available rumen datasets. Thus, our dataset substantially improves the coverage of rumen microbial genomes in the public databases and represents a valuable resource for biomass-degrading enzyme discovery and studies of the rumen microbiome.

## Abstract (web summary)

Microbes in the cow rumen are crucial for the breakdown of plant material. Here, Stewart et al. assemble over 900 bacterial and archaeal genomes from the cow rumen microbiome, revealing new species and genes encoding enzymes with potential roles in carbohydrate metabolism.

## Introduction

Cattle and other food-producing ruminants are of vital importance for human food security. The FAOSTAT database from 2013 reports over 296 million beef cattle and 273 million dairy cattle worldwide, and a further 468 million milk-producing ruminants (buffalo, goat and sheep). Therefore, understanding how ruminants convert their food into energy, and subsequently milk and muscle protein, is of obvious importance. If we can improve the efficiency of food digestion by ruminants, we may be able to produce more food while using fewer resources, a key aim of improving global food security.

The rumen contains a microbial ecosystem in which a dense and complex mixture of bacteria, archaea, protozoa and fungi convert carbohydrates to short-chain, volatile fatty acids (VFAs). Despite tremendous industrial and scientific interest, the rumen remains an under-characterized environment, containing many microbial species and strains that have not been cultured. In 2011, Hess et al.^1^ found that only 0.03% of their assembled rumen metagenome had hits to sequenced organisms. Despite the fact that many thousands of sequenced bacterial genomes have been deposited into public repositories since then, metagenomic sequencing of the rumen still produces highly novel sequences, which are significantly divergent from public collections^2,3^. The microbes within the rumen are also of interest to the biofuels and biotechnology industries, and metagenomics enables the identification of novel proteins of industrial interest^4^.

Metagenomic binning is a bioinformatics technique that enables near-complete microbial genomes to be assembled directly from metagenomic sequencing data. Tyson et al.^5^ were the first to recover near-complete genomes from a metagenome, in this case a natural acidophilic biofilm, and Hess et al.^1^ were the first to apply binning techniques to the rumen, recovering 15 microbial genomes with >60% completeness. Since then, while some rumen metagenomic studies have been published^2,6^, only Svartström et al.^7^ attempted metagenomic binning, recovering 99 metagenome-assembled genomes (MAGs) from just six moose samples. Additionally, Parks et al.^8^ report almost 8000 MAGs binned from over 1500 public datasets (which includes four rumen datasets).

Another powerful tool for the culture-free recovery of near-complete microbial genomes from complex environmental samples is Hi-C-based proximity-guided assembly. The Hi-C^9,10^ method cross-links DNA molecules that are in close physical proximity within intact cells. Followed by enzymatic digestion, proximity ligation and sequencing, this method yields paired reads that encapsulate ultra-long-range genomic contiguity, capturing interactions between multiple chromosomes as well as plasmids with their host genomes. These paired reads only link DNA molecules that co-existed in the same cell, and therefore can be used for metagenomic deconvolution and assembly.

Here we report the assembly of 913 near-complete and draft bacterial and archaeal genomes from a large rumen metagenomic sequencing study involving 43 Scottish cattle. We show that the addition of these genomes drastically improves our ability to quantify the taxonomic structure of the rumen microbiome, and many of the resultant genomes encode novel carbohydrate-active enzymes (CAZys) that represent candidates of potential use in the biofuels and biotechnology industries. We go on to characterize the polysaccharide utilization loci (PUL) of some of the genomes, identifying genomic patterns associated with particular carbohydrate substrates. Finally, by examining some of the less characterized genomes, we identify 31 genomes from the order *Erysipelotrichales*, members of which are now postulated to play an important role in animal physiology and disease^11^.

## Results

### 913 draft microbial genomes assembled from the cow rumen

We produced 768 Gb of Illumina sequencing data from 42 rumen microbiomes of Scottish cattle, carried out a metagenomic assembly of each sample individually and all samples collectively, creating a set of dereplicated putative genome bins with estimated completeness ≥80% and estimated contamination ≤10%. Our analyses resulted in 850 MAGs. The distribution of the 850 MAGs across the 42 metagenomic sample can be seen in Supplementary Data 1.

In addition, we sequenced a 43^rd^ sample on the Illumina platform and used Phase Genomics’ ProxiMeta Hi-C technology^10^ to cluster assembled contigs into genomes. Our Hi-C analysis resulted in a further 63 draft genomes with completeness ≥80% and contamination ≤10%. Hereafter, these two sets of genomes are referred to as RUGs (Rumen Uncultured Genomes) and hRUGs (Hi-C Rumen Uncultured Genomes), respectively.

Bowers et al.^12^ recently defined high-quality draft MAGs as having completeness >90% and contamination <5%, and 491 of our genomes meet these criteria; 215 are >95% complete, with <5% contamination, and 30 genomes have >97% completeness and 0% contamination.

**Fig. 1:**
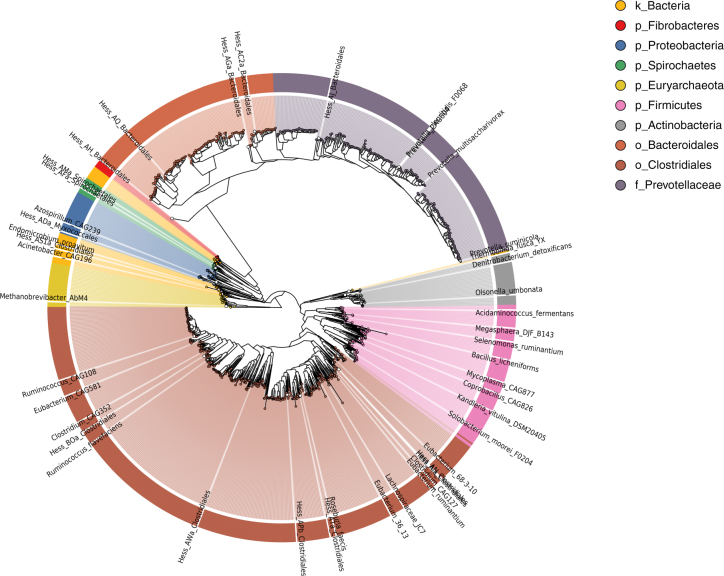
Phylogenetic tree of 913 draft genomes from the cow rumen, and closely related public genomes including 15 binned genomes from Hess et al^1^. Coloured circles represent the RUGs. White circles represent public genomes and have corresponding labels

Supplementary Data 2 describes the assembly characteristics and predicted taxon of each genome, and Fig. 1 shows a phylogenetic tree of the draft genomes alongside selected public genomes and the 15 binned genomes from Hess et al^1^. Supplementary Data 3 shows a linear representation of the same tree. Supplementary Data 4 shows the results of a comparison of the draft genomes with public genomes using MinHash^13^ signatures, and Supplementary Data 5 provides summary results comparing the RUG proteomes with UniProt TrEMBL^14^. All of the above were used to predict the most likely taxon of each genome (see Methods).

Several large clades are visible in Fig. 1. The tree is dominated by two large clusters representing *Clostridiales* and *Bacteroidales*, a significant cluster of the latter representing *Prevotellaceae*. Smaller clades represent the *Proteobacteria*, *Archaea*, *Actinobacteria*, *Spirochaetes* and *Fibrobacteres*. The remaining nodes and branches represent miscellaneous bacteria.

We can confidently resolve seven of the RUGs to species. RUG346 is a strain of *Bacillus licheniformis*, RUG287 a strain of *Kandleria vitulina*, RUG405 a strain of *Acidaminococcus fermentans*, RUG618 a strain of *Megasphaera *sp. (most similar to strain *DJF_B143*), RUG133 a strain of *Bifidobacterium merycicum* and RUG664 a strain of *Streptococcus equinus*. *Bacillus licheniformis* encodes both hemicellulolytic and cellulolytic enzymes^15^ as well as serine proteases and other important enzymes. *Kandleria vitulina*, relatively recently renamed^16^, is a little-studied organism, although the family *Erysiopelotrichaceae* (of which it is a member) has been positively correlated with milk yield^17^ and negatively correlated with methane emissions^18^. *Acidaminococcus fermentans* is a Gram-negative diplococcus that uses citrate as an energy source, producing hydrogen and hydrogen sulphide^19^, which may explain its association with methane production in cattle^2^ (methanogens convert H_2_ with CO_2_ to CH_4_). *Acidaminococcus fermentans* has also been shown to decrease the accumulation of tricarballylate, a toxic end product of ruminal fermentation, by oxidizing *trans*-aconitate^20^. *Megasphaera* spp. have been found in cattle, sheep and other ruminants. *Megasphaera elsdenii*’s ability to produce a variety of VFAs is of interest to the chemical industry^21^. Supplementation of the diet of dairy cows with *M. elsdenii*, which utilizes lactate as an energy source, has potential benefits for energy balance and animal productivity^22^. *Bifidobacterium merycicum* was first isolated from the rumen in 1991^23^, has been shown to ferment starch with the production of acetic and lactic acid^24^ and has been implicated in acidosis^25^. As the name suggests, *S. equinus* was first isolated from the horse; however, the related *S. bovis* is considered one of the key lactic acid-producing bacteria in the rumen, and has also been associated with acidosis^26^. Finally, RUG422 is a strain of *Thermobifida fusca*^27^, a likely contaminant from soil during grazing. We include it here for completeness and to improve the coverage of *Thermobifida* genomes in the public databases.

Of the remaining 906 RUGs, 158 were resolved to at least Genus, 416 to at least Family, 841 to Order, 845 to Class, 895 to Phylum and 906 to Kingdom. Twenty-eight of the RUGs represent archaea, and Supplementary Data 6 shows these in the context of 597 public archaeal genomes. We predict that all represent methanogens due to their position in the tree. In each case, the closest matched organisms represent the most abundant and metabolically active methanogens in ruminants^28^. Twenty-five of the RUGs cluster with other *Methanobrevibacter* species, with similarity to known ruminant methanogens such as *Methanobrevibacter ruminantium*^29^, *Methanobrevibacter millerae*^30^ and *Methanobrevibacter boviskoreani* (previously isolated from the rumen of Korean cattle^31^). RUG779 and hRUG898 are most closely related to *Candidatus* *Methanomethylophilus*, a methanogen originally isolated from human faeces that contains genes necessary for methanogenesis from methanol and trimethylamine, dimethylamine and monomethylamine^32^. RUG761 clusters closely with several *Methanosphaera* species. *Methanosphaera* strain WGK6, which RUG761 is most closely related to, was originally isolated from kangaroos^33^, and like the closely related *Methanosphaera stadtmanae*^34^, lacks many enzymes common to methanogens, and relies on acetate for synthesis of ATP.

Of the RUGs resolvable to phylum level, *Firmicutes* dominated (50%), followed by *Bacteroidetes* (36%), *Actinobacteria* (3.5%), *Proteobacteria* (3.1%), *Euryarchaeota* (3.1%) and *Spirochaetes* (1%), representing in general the most dominant microbial phyla identified in the rumen. The distributions of phyla across the MAGs and the Hi-C genomes are very similar.

### Thousands of novel CAZys

The CAZy database^35^ defines six classes of enzyme involved in carbohydrate metabolism. Glycoside hydrolases (GHs) hydrolyse the glycosidic bonds of complex carbohydrates and, within microbes, often assist in the degradation of cellulose, hemicellulose and starch; glycosyl transferases catalyse the formation of glycosidic bonds, utilizing sugar phosphates as donors, and transferring a glycosyl group to a nucleophilic group (together these two classes of enzyme form the major machinery for the breakage and synthesis of glycosidic bonds). Polysaccharide lyases (PLs) cleave glycosidic bonds in polysaccharides; and carbohydrate esterases (CEs) catalyse deacylation of polysaccharides. Finally, the auxiliary activities (AAs) class within CAZy describes a number of enzymes that act in conjunction with the first four classes, and the carbohydrate-binding (CB) modules describe proteins that adhere to carbohydrates.

The 913 RUGs contain 1,979,391 predicted proteins. These were searched against the CAZy database using dbCAN and filtered using suggested cut-offs^36^ (Supplementary Data 7). A total of 69,678 sequences were predicted to have at least one carbohydrate-active function. The proteins were compared to nr, env_nr, m5nr^37^, UniProt TrEMBL^14^ and the Hess et al.^1^ gene predictions to check for novelty (Supplementary Data 8), and against Pfam^38^ to detect other domains (Supplementary Data 9). Of the 69,678 proteins, only 6061 (8.7%) had a highly similar match in any of the above databases (⩾95% identity), indicating that 63,617 of our predicted carbohydrate-active proteins can be considered novel. To detect redundancy in our novel protein set, we clustered the protein sequences using CD-HIT at 99%, 95% and 90% identity, producing 51,117, 44,171 and 41,185 clusters, respectively.

**Fig. 2:**
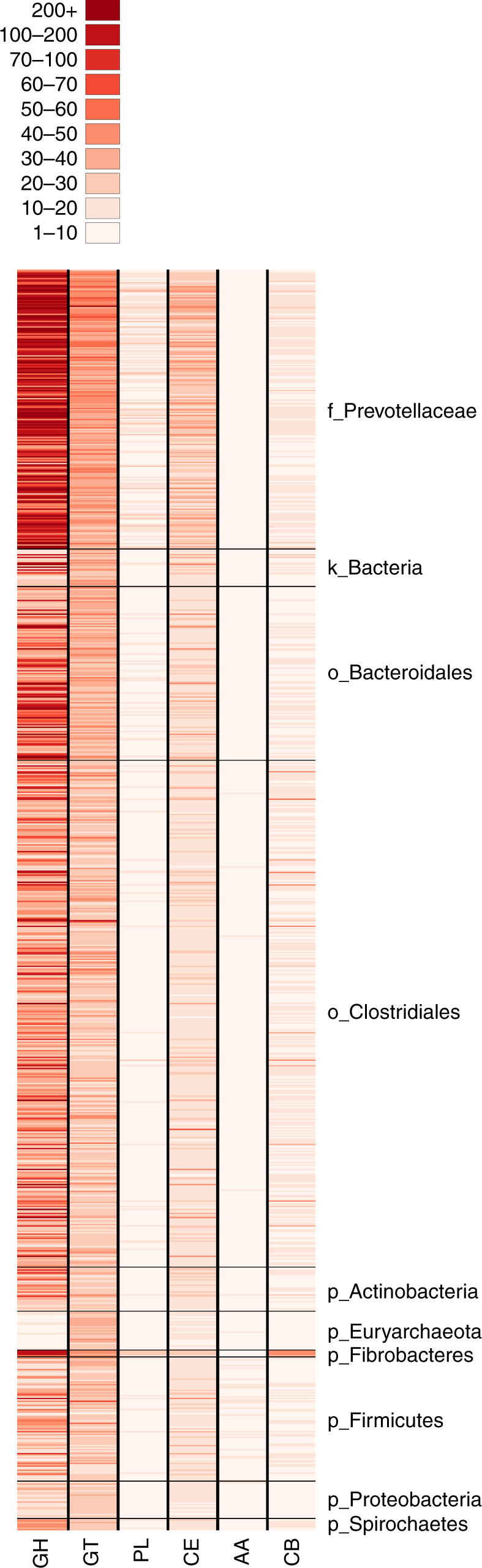
Distribution of carbohydrate-active enzyme classes across the 913 RUGs. GH glycoside hydrolase, GT glycosyl transferase, PL polysaccharide lyases, CE carbohydrate esterases, AA auxiliary activities, CB carbohydrate binding. White = absent, dark red = abundant

In total, and including proteins with multiple domains, the RUGs contain 40,140 GHs, 19,722 glycosyl transferases, 1121 PLs, 9119 CEs, 154 proteins with AAs and 2545 CB proteins. The distribution of these enzymes across the 913 RUGs can be seen in Fig. 2 and Supplementary Data 10. GHs and glycosyl transferases (GTs) are enriched in the *Prevotellaceae* and other *Bacteroidales*, *Fibrobacteres* and some of the *Clostridiales*, while being largely absent from the *Archaea*, and *Proteobacteria*.

**Fig. 3:**
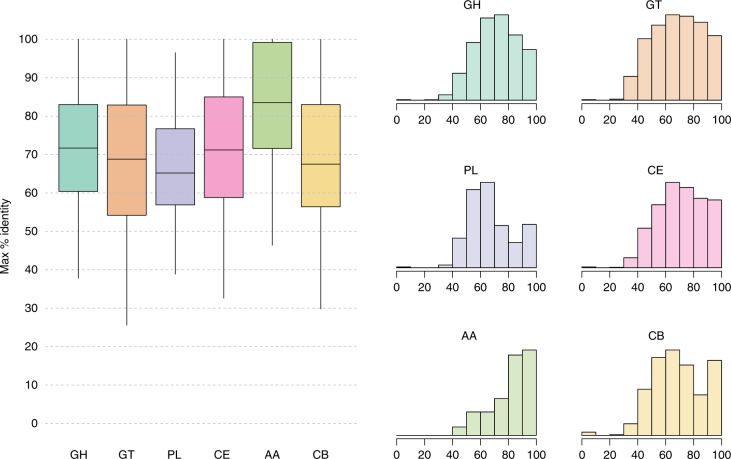
Distribution of the maximum percentage identity of the RUG proteins against five public databases for six classes of carbohydrate-active enzymes. GH glycoside hydrolase, GT glycosyl transferase, PL polysaccharide lyases, CE carbohydrate esterases, AA auxiliary activities, CB carbohydrate binding

To understand how different the RUG proteins are from those in the public databases, we plotted the percentage amino-acid identity of the best hit for each CAZy enzyme class (Fig. 3). On average, the predicted GHs, GTs, PLs, CEs and CB proteins are between 65 and 72% identical at the amino-acid level with current publicly available sequences. The AAs class are more conserved, with a median amino-acid identity around 83%.

We also investigated the ability of any of the RUGs to produce cellulosomes, multi-enzyme complexes responsible for the degradation of lignocellulosic biomass^39^. A key component of the cellulosome is a scaffoldin protein, which acts as a scaffold for the complex. The scaffoldin protein is characterized by multiple, repeated cohesin domains that the members of the cellulosome bind to^40^. There are 15 RUGs that contain proteins with multiple predicted cohesin domains: hRUG867 has one protein with five cohesin domains and another with two; both RUG167 and RUG148 encode proteins with six cohesin domains, and RUG738, RUG783 and RUG007 encode proteins with five. RUG640 encodes one protein with three cohesin domains, a further seven RUGs contain proteins with two cohesin domains (RUG291, RUG074, RUG394, RUG606, RUG393, RUG425, RUG502) and RUG817 contains two proteins with two predicted cohesin domains. According to our phylogenetic tree (Fig. 1 and Supplementary Data 3) 9 of the 15 RUGs with potential for producing cellulosomes cluster closely with *Ruminococcus flavefaciens*, a known cellulosome-encoding species, and one of the most dominant cellulolytic bacteria in the rumen^41,42^. However, many of these RUGs show an average protein identity of only between 70 and 80% with *R. flavefaciens*, indicating that these genomes are significantly different to existing *Ruminococcaceae* at the protein level. Five of the remainder are classified as a members of the *Clostridiales* order, and RUG606 is classified as a member of the *Prevotellaceae* family.

### Analysis of PUL

CAZys are often organized in PUL^43^, which represent series of linked genes that encode all activities necessary for the binding and degradation of complex polysaccharides, and which may be specific to a particular substrate, or target multiple substrates. The presence of particular PUL can therefore be used to predict the substrate specificity of microbial strains. We examined members of the *Prevotellaceae* family for PUL by first searching for tandem susC-susD-like pairs, followed by a moving window search for enzymes involved in carbohydrate binding and metabolism^44^.

Using this method, we identified 1743 putative PUL from 203 of the RUGs. A summary can be found in Supplementary Data 11 and full details in Supplementary Data 12. One hundred and eighty-seven of 203 RUGs contained more than one PUL, and the number of PUL per genome varied between 37 and 1. RUG144 and RUG423 contain 37 and 35 PUL, respectively, and are closely related in the phylogenetic tree. These two, along with other RUGs with large numbers of PUL, cluster together with *Prevotella multisaccharivorax*^45^, an anaerobic Gram-negative bacterium known for being able to utilize multiple polysaccharides as substrates. This branch of the tree therefore may be a fertile hunting ground for efficient and novel carbohydrate-active enzymes and microbes. A second clade of *Prevotella* RUGs with multiple PUL cluster around *Prevotella ruminicola*, one of the most abundant microbes cultured from the ruminant gut. The RUGs in this clade tended to have fewer PUL than those clustered with *P. multisaccharivorax*, suggesting a lower polysaccharide degradation potential. Enzymes present in PUL associated with *P. multisaccharivorax* include glucosidases, fucosidases, galactosidases, xylanases, arabinases, cellulases, levanases and mannosidases; and enzymes present in PUL associated with *P. ruminicola* include pectate lyases and pectin esterases, glucosidases, arabinases, galactosidases and mannosidases. These results suggest that both clades make use of multiple substrates, with a large overlap but some substrates unique to each clade.

We counted the occurrence of particular enzymes and the number of unique PUL they were associated with, and this can be seen in Supplementary Data 13. The most common enzymes were β-glucosidase and α-glucosidase involved in the breakdown of cellulose and starch, respectively, followed by β-galactosidase, which is involved in the breakdown of lactose into monosaccharides. Lactose is a major component of whey, which is sometimes fed to cattle, and is utilised by rumen bacteria during fermentation. Other common enzymes include arabinanase involved in the breakdown of arabinan, a component of sugar beet, a common dietary ingredient for cattle; xylanases involved in the breakdown of hemicellulose and plant cell walls; levanases which are part of the fructan production pathway; and also enzymes involved in the breakdown of pectins and mannans.

Supplementary Figure 1 shows a range of PUL. PUL41 (Supplementary Figure 1A) is representative of the most common PUL in the dataset—a single susC/D pair, and 526 of the PUL we predicted have this simple configuration. Svartström et al.^7^ discovered a similar result in the moose rumen, and speculated that some of the surrounding unclassified genes may be as-yet-undiscovered classes of CAZys. PUL 416, 1060, 1118 and 2240 (Supplementary Figure 1B–E) all show a similar configuration and are associated with xylan degradation, as they contain at least one copy of endo-1,4-β-xylanase. These PUL are characterized by a GH67-GH35-susC-susD-unc-GH10 pattern on one strand, preceded by a GH43-GH10 pattern on the opposite strand. The four PUL here show slight differences, but the pattern is obvious across all four PUL and may represent a configuration optimized for xylan degradation. PUL2240 is of further note as it is identical in structure to 'Predicted PUL 6' from 106_bin21 of Svartström et al^7^. Finally, two PUL with an identical configuration were identified as being involved in pectin degradation, both of which contain a pectate lyase and pectinesterase protein (Supplementary Figure 1F–G).

### Expansion of the *Erysipelotrichales* order

Of the RUGs that checkM could only resolve to the *Bacteria* kingdom, we noticed that 31 of them had significant numbers of protein hits to the *Solobacterium*, *Coprobacillus* and *Kandleria* genera, which are all members of the Erysipelotrichales order. Members of the *Erysipelotrichales* have been isolated from the human^11^, mouse^46^ and insect gut^47^ and pig manure^48^. We hypothesized that some of the 31 RUGs may represent new clades within *Erysipelotrichales* . The 31 genomes were placed into the microbial tree of life using PhyloPhlAn (Supplementary Data 14), and the sub-tree representing *Erysipelotrichales* is shown in Supplementary Data 15.

Sixteen of the RUGs cluster closely with *Solobacterium moorei*, first isolated from human faeces^49^. On the basis of the tree and the low average protein identity, we propose that these RUGs are putative novel *Solobacterium* species, and we have named them accordingly in Supplementary Data 16 ('uncultured rumen *Solobacterium*'). RUGs 521 and 747 sit between the *Solobacterium*/*Bulleidia* clade and that of *Holdemania filiformis*; therefore, both of these genomes may represent novel genera within the *Erysipelotrichaceae* family, although more data are required on their physical and phenotypic properties before this can be determined—we therefore propose the name 'uncultured rumen *Erysipelotrichaceae*'. A further ten RUGs cluster quite separately from the other members of the *Erysipelotrichaceae*, with only one public genome, *Coprobacillus *sp.* CAG_826 *(another MAG from the human microbiome), nearby. The coprobacilli are a diverse and somewhat ill-defined group. Indeed, many former members of the *Erysipelotrichaceae* have been suggested to be members of a new family, *Coprobacillaceae*^47^, within the *Erysipelotrichales* Order, and the above RUGs cluster more closely with this clade. We therefore propose the name 'uncultured rumen *Coprobacillaceae*' for these RUGs. Finally, three RUGs cluster closely with *K. vitulina*. RUG287 appears very closely related to four other sequenced *K. vitulina* genomes and we propose the name *Candidatus K. vitulina* strain RUG287 for this organism. RUGs 319 and 246 cluster separately, and we propose names *C. Kandleria *sp. strain RUG319 and *C. Kandleria *sp. strain RUG246 for these organisms.

Overall, the addition of 31 MAGs to this branch of the tree of life represents a significant expansion of the *Erysipelotrichaceae* and *Coprobacillaceae* families in reference databases.

### Improved metagenomic classification

**Fig. 4:**
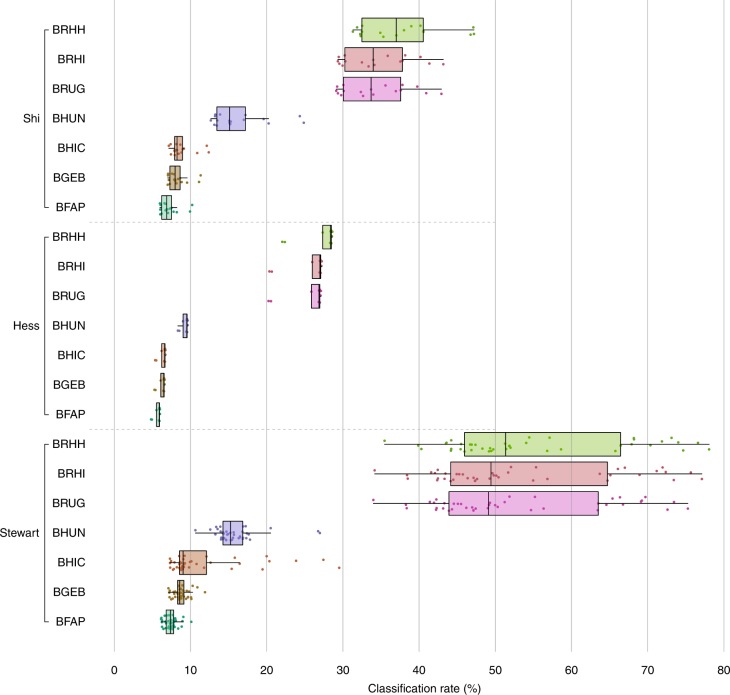
Classification rate for three datasets against various Kraken databases. BFAP bacterial, archaeal, fungal and protozoan genomes from RefSeq, BGEB BFAP + 1003 GEBA genomes, BHIC BFAP + 63 hRUG genomes, BHUN BFAP + 410 genomes from the Hungate 1000 project, BRUG BFAP + 850 RUG MAGs, BRHI BFAP + all 913 genomes from this study, BRHH BFAP + 913 RUGs + 410 Hungate 1000 genomes. Addition of rumen-specific RUGs or Hungate 1000 genomes has the most dramatic effect

Reads from this study, Hess et al.^1^ and Shi et al.^6^ were taxonomically assigned to seven different sequence databases using Kraken^50^ (see Methods). The base reference database consisted of bacterial, archaeal, fungal and protozoan genomes from RefSeq^51^ (BFAP). Then, each of GEBA^52^ (BGEB), the hRUG genomes (BHIC), the Hungate 1000^53^ genomes (BHUN) and the RUGs (BRUG) were added; finally, all hRUGs and RUGs (BRHI) and all hRUGs and RUGs plus Hungate were added (BRHH). The effects on classification rate can be seen in Fig. 4. In all three datasets, the base RefSeq database classifies fewer than 10% of the reads, and addition of the GEBA genomes has only a marginal effect. Addition of the Hungate 1000 genomes increases classification around twofold; however, addition of the RUGs increases the classification rate considerably, by around sevenfold in our own data, and by around fivefold in the two other publicly available rumen metagenome datasets; the addition of the Hi-C genomes alone has only a marginal effect in the two public datasets, yet has a larger effect in our own data; and addition of both sets of rumen genomes performed best in all datasets; however, the addition of the 410 Hungate 1000 genomes only improved on the RUGs by 2–4%. Overall classification rates were improved by fivefold to sevenfold when adding rumen-specific bacteria and archaea from this study and the Hungate 1000 project, with classification rates in some cases approaching 80% against our own data.

The classification rate for Hess et al.^1^ is noticeably lower than the other two datasets. We note that both our data and the Shi et al.^6^ data come from rumen fluid, whereas the Hess et al.^1^ data are specifically switchgrass-associated, raising the possibility that these two microbiomes are significantly different. We re-classified all samples using Centrifuge^54^, an efficient metagenomic classifier capable of indexing the entirety of nt. While the Kraken databases contain only microbial sequences, the nt database contains non-redundant sequences from all sequenced clades of life, and may be informative about the presence of non-microbial DNA in the Hess et al.^1^ samples. However, classification results against nt need be interpreted with care as many entries are mis-labelled or contain contamination. These analyses identified a number of factors, which may explain the lower classification rates in the Hess et al.^1^ data. First, the Hess et al.^1^ data have reads of length 100 bp, whereas both our and the Shi et al.^6^ data are reads of length 150 bp. The shorter read lengths may influence Kraken’s ability to find matches in the database. Second, as may be expected, the Hess et al.^1^ data included more reads originating from wheat and switchgrass (Supplementary Figure 2). Finally, *Prevotella* were generally more abundant in our data and the Shi et al.^6^ data, and *Fibrobacter* more abundant in the Hess et al.^1^ data (Supplementary Figure 3). *Fibrobacter succinogenes* is a ruminant bacterial species that is known to associate with fibrous plant material^42^.

### Analysis of Hi-C clustering

We have the opportunity to analyse the benefits of using Hi-C clustering over and above single-sample metagenomic binning. As previously described, the Hi-C analysis produced 63 genomes with ≥80% completeness and ≤10% contamination. We used the same assembly as input to MetaBAT2 and carried out metagenomic binning. This resulted in 76 genomes using the same cut-offs (Supplementary Data 17). We hypothesized, however, that the Hi-C genomes may contain more copies of genetic elements that do not conform to the origin cell’s genomic norms—for example, plasmids often exist in multiple copies, and often have a different base-composition to that of the core genome. As binning algorithms such as MetaBAT use both base-composition and coverage, plasmids are often missing from MAGs.

Boxplots of the coverage of each contig within each bin can be seen in Supplementary Figure 4. There are no gross differences of the median coverage statistics between the Hi-C and SPAdes/MetaBAT clusters; however, there is a very noticeable difference in the range, with the Hi-C clusters showing a far greater range of coverage values. This is to be expected—MetaBAT by design clusters contigs that are similar in coverage value, whereas Hi-C clustering has no such limitation.

To look for the presence of multi-copy plasmids, we extracted from each set of genomes (the 63 Hi-C genomes and the 76 SPAdes/MetaBAT genomes) those contigs that had >2× the mean average coverage for their respective genome. This resulted in 243 contigs from the Hi-C genomes and 37 from the SPAdes/MetaBAT genomes. These were searched against the nt database using NCBI BLAST+^55^. After filtering out short hits, there were no contigs in the SPAdes/MetaBAT set that could be annotated as 'plasmid', whereas there were ten contigs (from ten different genomes) in the Hi-C set that could (Supplementary Data 18).

Supplementary Figure 5 shows a heatmap of the number of Hi-C links between the 10 putative plasmid contigs and the 63 Hi-C genomes. Two of the contigs, NODE_52225_length_2785_cov_42.9597 and NODE_49376_length_2919_cov_46.8415, have BLAST hits to plasmid pRUMAL02, originally discovered in *Ruminococcus albus* and found to code for dockerin-containing proteins^56^. These two contigs appear to be shared across four of the hRUG genomes, three in the large *Clostridiales* cluster (hRUG877, hRUG853, hRUG861), and a fourth which clusters closely with *R. flavefaciens* (hRUG867). While very preliminary in nature, these results suggest that plasmids encoding proteins responsible for carbohydrate degradation may be shared between these different species.

## Discussion

Beef cattle, dairy cattle and other milk-producing ruminants provide food and nutrition to billions of people worldwide. It is the rumen microbiome that is largely responsible for the extraction of energy and nutrients from the diverse diets fed to farmed ruminants, and it is vital that we understand the structure and function of this important microbiome. Despite this importance, the rumen microbiome remains to a large extent under-characterized, and very few rumen microbial genomes exist in the public databases.

The RefSeq database now contains well over 75,000 prokaryotic genomes^57^, driven by decreasing sequencing costs^58^, improvements in bioinformatics techniques^59^ and large projects focused on specific environments.

In this study, we show that current RefSeq genomes are very poor at aiding the classification of reads from the rumen microbiome, and only by sequencing microbes specifically from the rumen do we begin to see improvements in classification, with the genomes from this study and that of the Hungate 1000 improving classification rates fivefold to sevenfold. However, the greatest impact on classification came from the 850 RUGs, which had a dramatic effect, increasing classification rates up to sevenfold higher than the base BFAP database. Adding Hungate 1000 on top of the RUGs increased rates by a further 2–4%. Of course, the Hungate 1000 collection has other advantages—being cultured, they can be studied in vitro and in vivo, and are classified to the species level. Nevertheless, publication of the 913 MAGs presented here could transform the way we interpret rumen metagenomics data, a field where 50–80% read classification rates have rarely, if ever, been achieved.

We used two different approaches for the recovery of genomes from metagenomes—metagenomic binning and Hi-C clustering. The Hi-C data provide direct physical evidence that contigs originate from the same genome, yet because they are used to cluster an assembled metagenome, they suffer from some of the same issues as any short-read assembly— collapsed repeats and the loss of complex regions that simply do not assemble well. Nonetheless, the Hi-C genome clusters are at least as good as the MAGs, and in general have lower contamination values.

The read classification rates for the Hi-C data require further examination. While the Hi-C genomes considerably improved (in many cases) classification rates against our own data (from other Scottish cattle), they had only a marginal effect on the two public metagenomes. This would suggest that the Hi-C genomes are population or environment specific, or that they were not numerous enough in this study of one sample. Increasing classification rates for particular populations is important - and in seven of our samples, the Hi-C genomes increased classification rates by more than the 410 Hungate 1000 genomes, a collection of cultured ruminant microbe isolates collated by a global network.

The performance of the Hi-C data can be contrasted with that of the 850 RUGs, which improved the read classification rates of all samples from all three datasets. The RUGs were created from 42 single-sample assemblies, and a 42-sample co-assembly (followed by dereplication) created from 768 Gb of data, whereas the Hi-C genomes were generated with only 41.7 Gb from a single individual. It is inevitable that the former contains more information, will reflect a more general consensus set of rumen microbes, and certainly will better represent low abundance microbes. There may also be an advantage to co-assembly, in that it assembles genomes present in multiple samples (and therefore more likely to be derived from core-microbiome members), compared to single-sample assemblies, which may preferentially assemble sample-specific genomes. On the other hand, individually assembling more samples using a method like Hi-C may increase the overall genome database of rumen microbes at a much-reduced amount of required sequencing and sampling.

There are now various methods for assembling MAGs direct from sequence data without the need for culture. Assembly and binning genomes from single samples results in genomes with lower contamination values; however, by definition, these genomes tend be biased towards more abundant microbes as the relative quantity of sequence data is not sufficient to assemble the low abundance members. Alternatively, assembly and binning of sequence data from a co-assembly of multiple samples produces genomes with higher contamination values, and often the accessory genome from multiple similar strains is co-assembled into a single genome. As CheckM only assesses the core genome, this can be missed. However, the advantage of the co-assembly approach is that a single assembly graph is created from all data, which means that lower abundance microbes are fully represented. Supplementary Data 19 shows the 850 RUGs sorted by average coverage, from lowest to highest. It is striking that almost all of the low abundance genomes come from the co-assembly. Best practice is therefore to combine single-sample assemblies with a co-assembly, followed by dereplication, which we have carried out in this paper. Finally, Hi-C clustering offers another way to produce MAGs. While we show here that Hi-C clustering does not necessarily produce more genomes than single-sample assembly binning, it does allow for contigs with higher than average abundance to be clustered into the host genome, and we present here some preliminary evidence that Hi-C clustering allows the clustering of plasmid sequences with their core genome, whereas single-sample assembly binning does not.

Eventually, if we are to fully understand the function of any microbiome, we must aim for 100% classification—in other words, to understand which genome each read comes from, and what functions those genomes encode. By assembling 913 genomes from our own dataset, and adding other rumen-specific genomes, some of our samples showed a classification rate close to 80%. Therefore, assigning taxonomies and functions to assemblies rather than the reads themselves could provide far greater insight. We have adopted a three-stage process in our microbiome research: stage 1 is discovery, simply sequencing what is there; stage 2 is association, correlating traits of interest with changes in the microbiome; and stage 3 is intervention, where we test microbial interventions to see whether we can alter those traits. From our work in this and previous studies^2^, it is clear that the rumen microbiome requires investment in discovery in order to maximize our potential to associate and intervene.

The rumen is of huge industrial interest due to its ability to release energy and nutrition from plant material. We show here that the 913 RUGs contain thousands of CAZys that differ significantly from existing representatives in the public domain. The RUGs contain over 69,000 proteins that are likely to be involved in carbohydrate metabolism, and which share on average only 60 to 70% amino-acid identity with similar protein sequences in the public domain. We identify 15 RUGs that potentially encode the machinery to produce cellulosomes, multi-enzyme complexes that have high cellulolytic activity and predict 1743 PUL from the *Prevotellaceae* family, one of the dominant saccharolytic families in the rumen, that contain proteins capable of binding and digesting multiple carbohydrate substrates. Several of the RUGs with the most PUL are related to *P. multisaccharivorax*, a bacterium capable of digesting several carbohydrate substrates, and these RUGs are likely to have large saccharolytic potential and be able to adapt to multiple diets and rumen environments. This paper represents the first *P*. *multisaccharivorax*-like genomes isolated from the rumen. We also identify RUGs with large numbers of PUL related to *P. ruminicola*.

The Erysipelotrichales are thought to have an increasingly important role in animal microbiomes. They have been found to be highly immunogenic and associated with multiple human diseases (reviewed by Kaakoush^11^). Shifts in the abundance of *Erysipelotrichaceae* have been associated with monensin application in cows^60^ (monensin is known to increase propionate in the rumen and improve energy availability) and high grain feeds in goats^61^, while studies in dogs show *Erysipelotrichaceae* are involved in the digestion of protein and energy production^62^. In this study, we identify and publish the sequence of 31 new members of the *Erysipelotrichaceae* (and closely related *Coprobacillaceae*) family. This represents a large expansion of our knowledge of these families' genomes, especially considering those isolated from the rumen.

As we and others have shown, metagenomic binning is a powerful technique for the recovery of complete and near-complete microbial genomes without the need to culture. However, the assemblies remain fragmented, and it would be beneficial to completely assemble entire chromosomes. New sequencing technologies such as that offered by Pacific Biosystems and Oxford Nanopore^63^ are now able to produce long reads at reasonable scale and cost, and hybrid approaches enable complete chromosomal assemblies from complex bacterial genomes^64^. There is every reason to expect that hybrid short-read and long-read sequencing will enable the complete end-to-end assembly of microbial chromosomes direct from metagenomic samples, and these approaches could revolutionize our understanding of complex microbiomes.

## Methods

### Metagenomic samples

Animal experiments were conducted at the Beef and Sheep Research Centre of Scotland’s Rural College (SRUC). The experiment was approved by the Animal Experiment Committee of SRUC and was conducted in accordance with the requirements of the UK Animals (Scientific Procedures) Act 1986.

The data were obtained from three cross breeds: Aberdeen Angus, Limousin and Charolais and one pure breed: Luing. The animals were offered two complete diets ad libitum consisting (g/kg DM) of either 500 forage to 500 concentrate or 80 forage to 920 concentrate. As previously described in Roehe et al.^3^, the animals were slaughtered in a commercial abattoir where two post-mortem digesta samples (approximately 50 mL) were taken immediately after the rumen was opened to be drained. DNA extraction was carried out following the protocol of Yu and Morrison^65^ and based on repeated bead beating plus column filtration. Illumina TruSeq libraries were prepared from genomic DNA and sequenced on an Illumina HiSeq 4000 by Edinburgh Genomics.

### Hi-C sample

Post-mortem rumen digesta from one beef cattle was collected in 125 mL screw cap container at the abattoir and transferred on ice. Then, the equivalent of 1.5 mL of sample was resuspended using 13.5 mL (10× volume or more) of 1% formaldehyde-phosphate-buffered saline (PBS) solution in a 15 mL Falcon tube. The sample was incubated at room temperature for 30 min with periodic mixing or vortexing followed by the addition of glycine to 1 g/100 mL or 125 mM final concentration. A second incubation was performed at room temperature for 15 min with periodic mixing or vortexing. The final step involved a series of spin down and rinses with PBS of the pellet. Briefly, the sample was spun down for 2 min at 6000 rpm, rinsed with PBS and spun down again (5 min at 6000 rpm), prior to removing the supernatant. The final pellet was kept frozen at −20 °C.

Cells were lysed with glass bead disruption in a detergent buffer, and DNA was extracted using the ZymoBiomics DNA Mini Kit (Zymo Research). For shotgun library creation, 100 ng was sheared to ~400 bp average insert length and used to create a library using the HyperPrep kit (KAPA Biosystems). Approximately 200 μL of solid material from the same rumen sample was crosslinked for Hi-C using standard protocols^9^ and split into two fractions. Each fraction was used to generate a Hi-C library using the proprietary ProxiMeta Hi-C protocol developed by Phase Genomics (similar Hi-C protocols have been published^9^). Each Hi-C sample was fragmented using either the Sau3AI or MluCI restriction enzymes prior to proximity ligation.

The shotgun and Hi-C libraries were sequenced on the Illumina HiSeqX platform, generating 150 bp paired-end reads. Sequencing of the shotgun library produced 139.6 million read pairs. Sequencing of the Hi-C libraries generated 86.2 million read pairs for the Sau3AI library and 59.3 million read pairs for the MluCI library.

### Bioinformatics

Adapters were trimmed from the Illumina data using Trimmomatic^66^ and the subsequent trimmed reads used as input for MEGAHIT^67^. A 42-metagenome co-assembly was carried out using options --kmin-1pass, -m 60e + 10, --k-list 27,37,47,57,67,77,87, --min-contig-len 300, -t 16. In addition, 42 single-sample assemblies were performed using idba_ud^68^ with the options --num_threads 16 --pre_correction --min_contig 300. BWA MEM^69^ was used to map reads back to the filtered assembly and Samtools^70^ was used to convert to BAM format. Script jgi_summarize_bam_contig_depths from the MetaBAT2 package was used to calculate coverage from the resulting BAM files.

Metagenomic binning was applied to both single-sample assemblies and the co-assembly using MetaBAT2^94^, with options --minContigLength 2000, --minContigDepth 2. Coverage values across the 42-sample dataset were taken into account. Single-sample binning produced a total of 4106 bins, and co-assembly binning produced a further 3253. All 7359 bins were aggregated and then dereplicated using dRep^71^. The dRep dereplication workflow was used with options dereplicate_wf -p 16 -comp 80 -con 10 -str 100. Only the highest scoring MAG from each secondary cluster is retained in the dereplicated set. For our dataset, 850 dereplicated MAGs were obtained, 699 from the single-sample assemblies and 151 from the co-assembly.

For the Hi-C sample, we trimmed adapter sequences from shotgun reads using BBDuk with options k = 23, ktrim = r, mink = 12, hdist = 1, minlength = 50, --tpe, --tbo. Next, we performed quality trimming of the reads using BBDuk and options qtrim = rl, trimq = 10, minlength = 50, chastityfilter = True. We then normalized read coverage using BBNorm with options target = 40, mindepth = 2. With this trimmed and normalized dataset, we performed a de novo shotgun assembly using metaSPAdes and default parameters^72^.

Using the Hi-C read datasets generated as described above, we trimmed each read to 75 bp to avoid discarding reads sequencing through a Hi-C junction. We mapped each read dataset (MluCI and SauIII) to the shotgun assembly described above using bwa aln^73^ while requiring perfect matches (option -n 0). We were able to map forward and reverse reads to different contigs for a total of 3,668,548 read pairs from the MluC1 dataset and 15,672,416 read pairs from the SauIII dataset. These reads could therefore be used to link contigs for deconvolution.

We performed deconvolution of the shotgun assembly into genomes using the proprietary ProxiMeta™ platform, which is similar to the previously described MetaPhase technique^9^. We filtered out all reads that were not properly paired, unmapped, non-uniquely mapped, had a MAPQ score <20, or were paired with a mate with an identical position. We filtered out contigs that were <2 kb in size, or which contained fewer than 10 restriction sites for the relevant enzyme. We combined datasets of the two restriction enzymes into a graph, and applied a normalization to the read counts connecting each pair of contigs by accounting for restriction site number of each contig. We clustered contigs into genome clusters using a proprietary Markov chain Monte Carlo-based algorithm based on their Hi-C linkages.

CheckM with options lineage_wf, -t 16, -x fa was used to assess the completeness and contamination of all bins. After filtering for completeness ≥80% and contamination ≤10%, we were left with 850 draft genomes from the 42 samples and a further 63 from the Hi-C analysis.

The predicted proteomes from each bin are produced as part of the CheckM process. The proteomes of each bin were compared to UniProt TrEMBL^14^ using Diamond^74^, and the top hit, length and percentage identity recorded. This allowed us to predict the most likely genus for each contig within each bin. Genera not within the Bacteria or Archaea lineages were flagged as problematic (*n* = 150), and contigs with these genera were removed from their respective bins. The resulting 'cleaned' bins were re-processed using CheckM, and the results can be seen in Supplementary Data 2. ETE3^75^ was used to expand the CheckM predicted taxon to a full taxonomy list. Our MAG annotation pipeline is available as a reproducible workflow^76^. Genomes and gene predictions were loaded into a Meta4 database^77^

The cleaned bins were compared, using MinHash sketches as implemented in Sourmash^78^, to 100,000 genomes in GenBank; 410 genomes from the Hungate 1000 project;^53^ and 1003 genomes from the GEBA project^52^. The results can be seen in Supplementary Data 4. The best hits from these analyses, combined with top hits from the UniProt searches above, were used to select publicly available genomes for comparison. This was a manual process. Initially a tree consisting of the 913 RUGs, the 410 Hungate 1000 genomes and genomes from the Uniprot results with >80% protein identity was calculated with PhyloPhlAn^79^ and visualized using GraPhlAn^80^ and FigTree. This initial tree, which relates the RUGs to their most closely related genomes, in combination with the outputs from CheckM, UniProt and Sourmash searches, allowed us to assign a new taxon to each of the bins, and these can be seen in Supplementary Data 2. The tree was subsequently re-drawn, with the Hungate 1000 genomes removed, and selected public genomes kept to help the viewer identify each clade. The updated taxonomic assignments and can be seen in Fig. 1.

Predicted proteins were compared to Pfam^38^ using pfam_scan.pl; the CAZy^35^ database using dbCAN;^36^ and to nr, env_nr, md5nr^37^ and the Hess et al.^1^ predicted proteins using Diamond^74^.

Reads were classified using Kraken, against six custom databases: BFAP, consisting of 7318 complete bacterial genomes, 229 fungal genomes, 585 archaeal genomes and 75 protozoan genomes (all from RefSeq); BGEB, consisting of the genomes from BFAP but with the additional 1003 genomes from the GEBA project;^52^ BHIC, consisting of BFAP + the hRUGs; BRUG, consisting of BFAP + the 850 RUGs; BHUN, consisting of the BFAP database plus 410 genomes from the Hungate 1000 project; BRHI, consisting of the BFAP database plus all 913 genomes presented in this study; and BRHH, consisting of BFAP, the 913 rumen genomes from this study, and the 410 Hungate 1000 genomes.

### PUL analysis

Prediction of PUL was carried out following a protocol similar to PULDB^44^. Using the data we had generated already (comparison of all protein predictions to Pfam and CAZy), we first identified susC/susD pairs in all assembled contigs. We then searched, using a simple moving window consisting of five protein predictions upstream and downstream, for proteins with a predicted homologue in the CAZy database. If one was found, the sliding window was moved in that direction, and the search repeated. The search ended when no more homologues from CAZy were found. The PUL were drawn using BioPython^81^ and GenomeDiagram^82^.

### Hi-C plasmid analysis

The same metaSPAdes assembly that was used for Hi-C clustering was also used for MetaBAT binning, using the single-sample protocol described above. Subsequently, after filtering for completeness ≥80% and contamination ≤10%, contigs with greater than twice their genome average were extracted for analysis. NCBI BLAST + version 2.4.0 was used to blastn search the contig sequences against the nt database downloaded on Friday 17th November 2017. Options to blastn were -outfmt '7 qseqid qlen sseqid sacc stitle slen qstart qend sstart send length evalue bitscore pident staxid' -evalue 0.01 -num_threads 16. High scoring pairs <500 bp and <10% of the query length were filtered out using awk. Hits that passed filter and contained the word 'plasmid' in the title are reported in Supplementary Data 18.

### Data availability

All raw sequence data have been submitted to the European Nucleotide Archive under project PRJEB21624. RUG and hRUG assembled genomes and proteomes are available from ENA and also from Edinburgh DataShare (DOI:10.7488/ds/2296) All other relevant data are available in this article and its Supplementary Information files, or from the corresponding author upon request.

## Electronic supplementary material

Supplementary Information

Description of Additional Supplementary Files

Supplementary Data 1

Supplementary Data 2

Supplementary Data 3

Supplementary Data 4

Supplementary Data 5

Supplementary Data 6

Supplementary Data 7

Supplementary Data 8

Supplementary Data 9

Supplementary Data 10

Supplementary Data 11

Supplementary Data 12

Supplementary Data 13

Supplementary Data 14

Supplementary Data 15

Supplementary Data 16

Supplementary Data 17

Supplementary Data 18

Supplementary Data 19
