# Supplementary material for: Assembly of 913 microbial genomes from metagenomic sequencing of the cow rumen
Source: Nat Commun. 2018 Feb 28;9:870. doi: 10.1038/s41467-018-03317-6 (PMC5830445; doi:10.1038/s41467-018-03317-6)
Supplement: Supplementary file 1 — Supplementary Information [file 41467_2018_3317_MOESM1_ESM.pdf]

Assembly of 913 microbial genomes from metagenomic sequencing of the cow rumen

Stewart *et al*

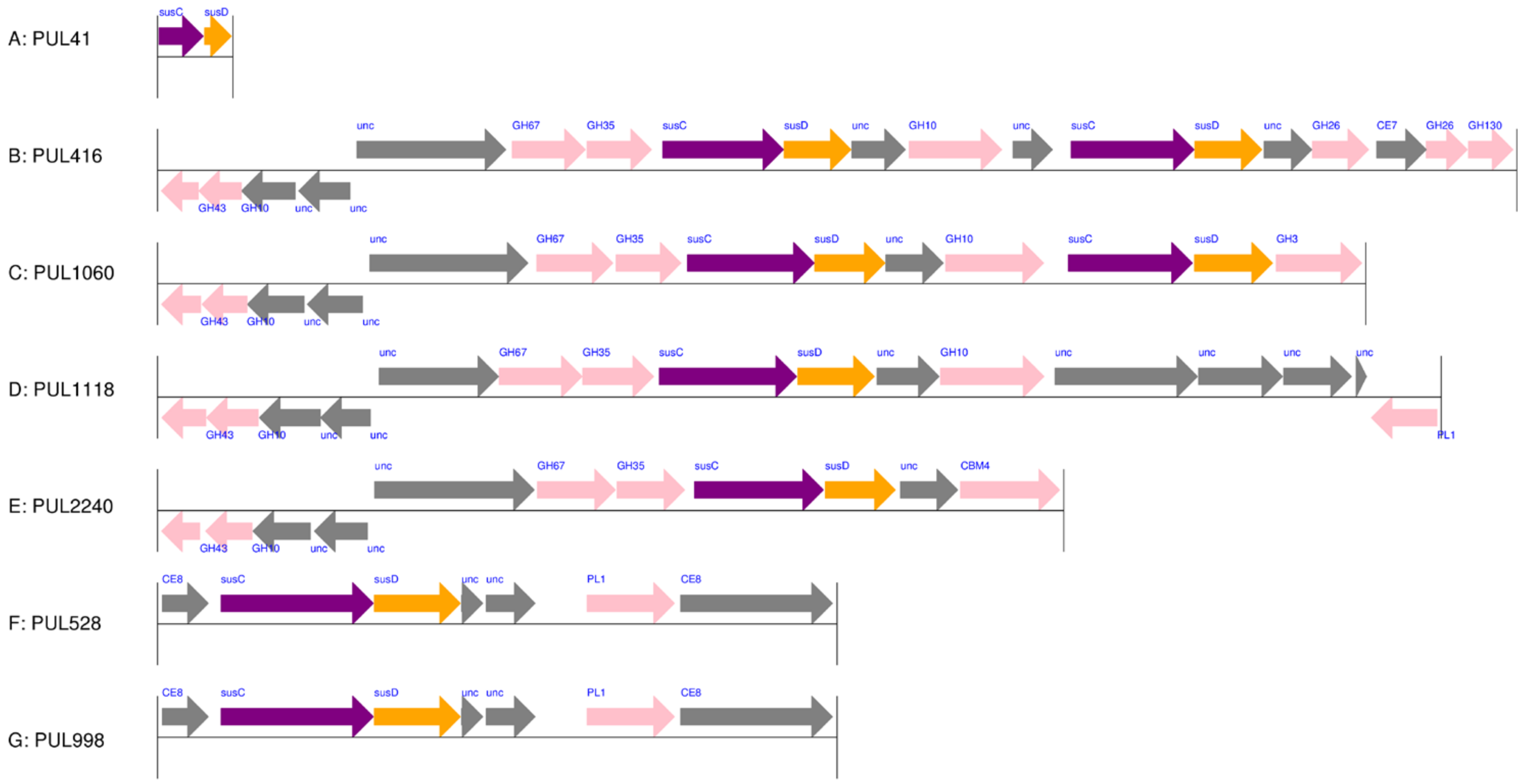

Supplementary Figure 1 A selection of predicted PULs. A: the most common, a simple susC/susD pair; B-E: a variety of PULs with similar configurations involved in xylan degradation; F-G: two identical PULs involved in pectin degradation

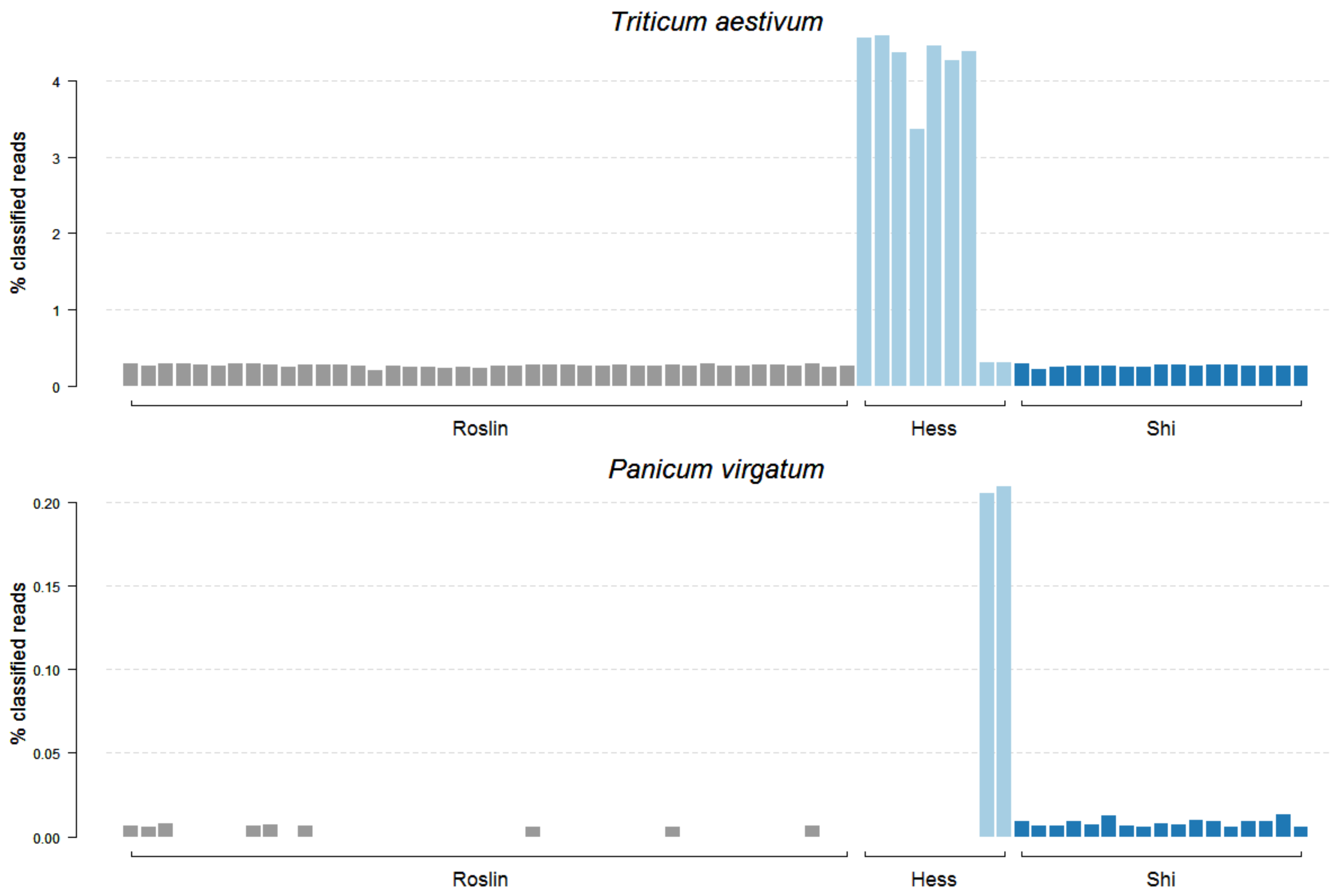

Supplementary Figure 2 Predicted contamination from plants in our data and the Hess and Shi data (using Centrifuge).

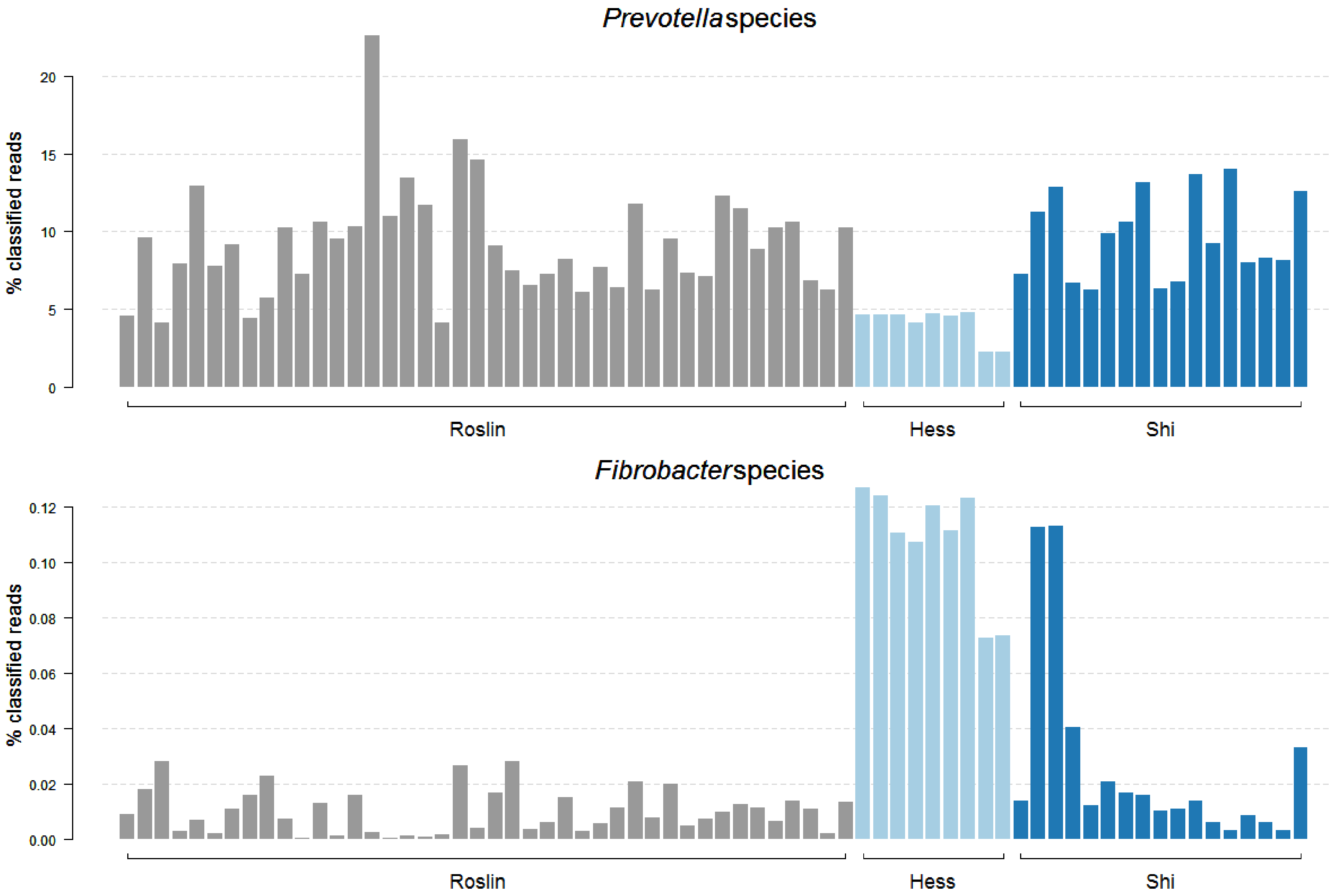

Supplementary Figure 3 Predicted distribution of *Prevotella* and *Fibrobacter* species in our data and the Hess and Shi data (using Centrifuge).

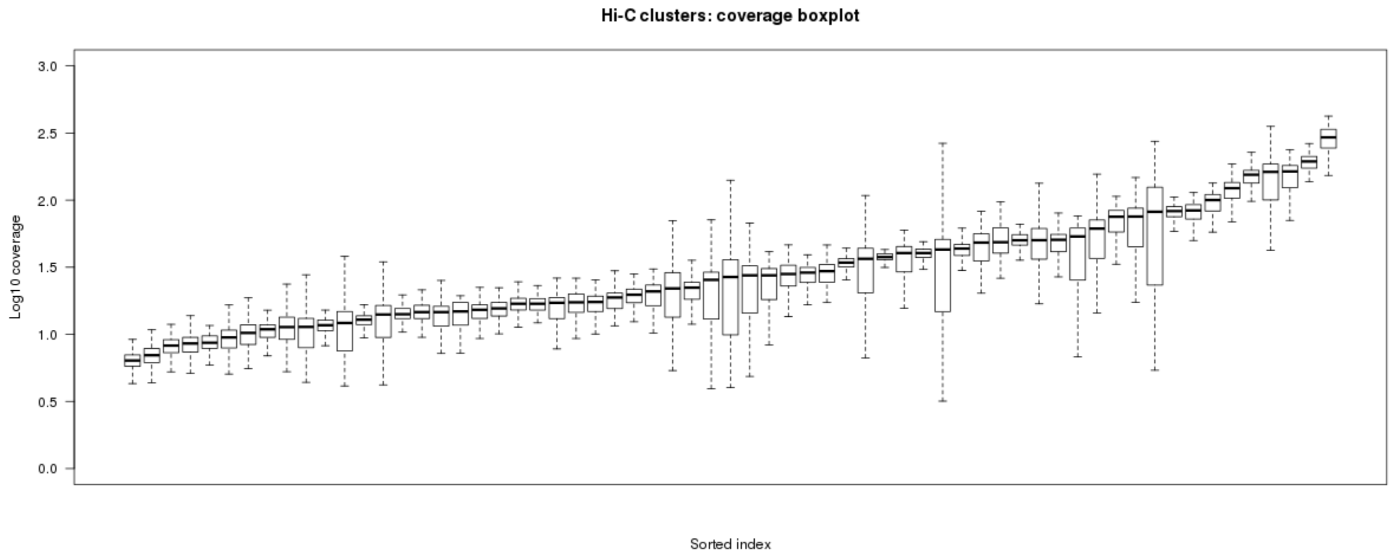

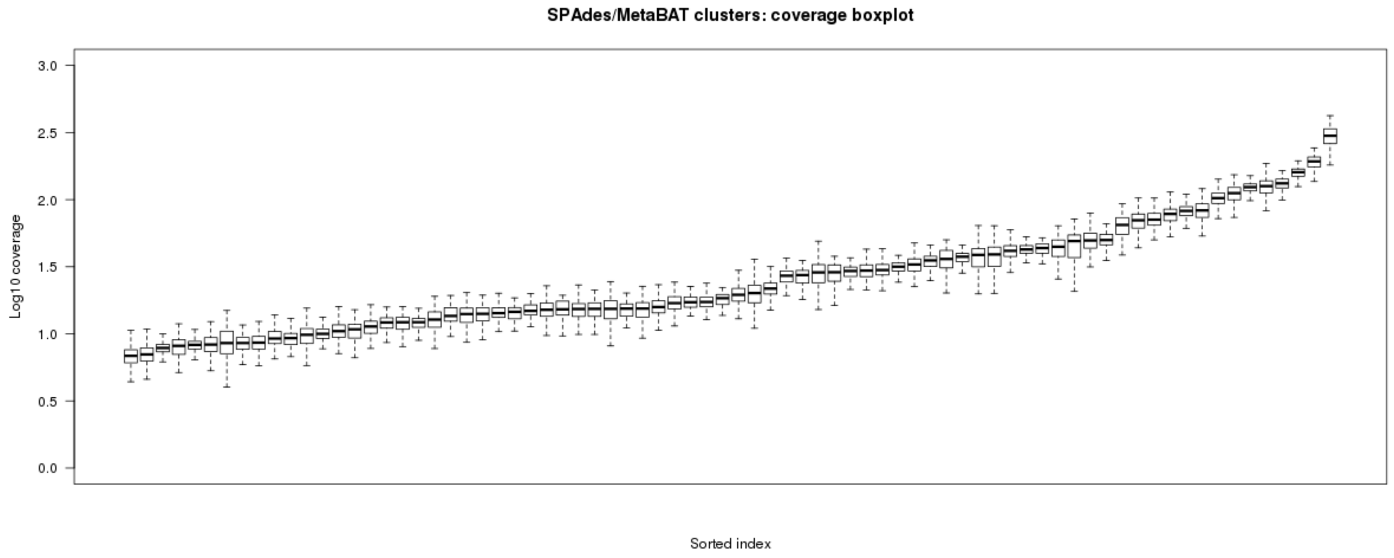

Supplementary Figure 4 Range of coverage values for contigs within bins from the 63 Hi-C genomes and the 76 metaSPAdes/MetaBAT genomes

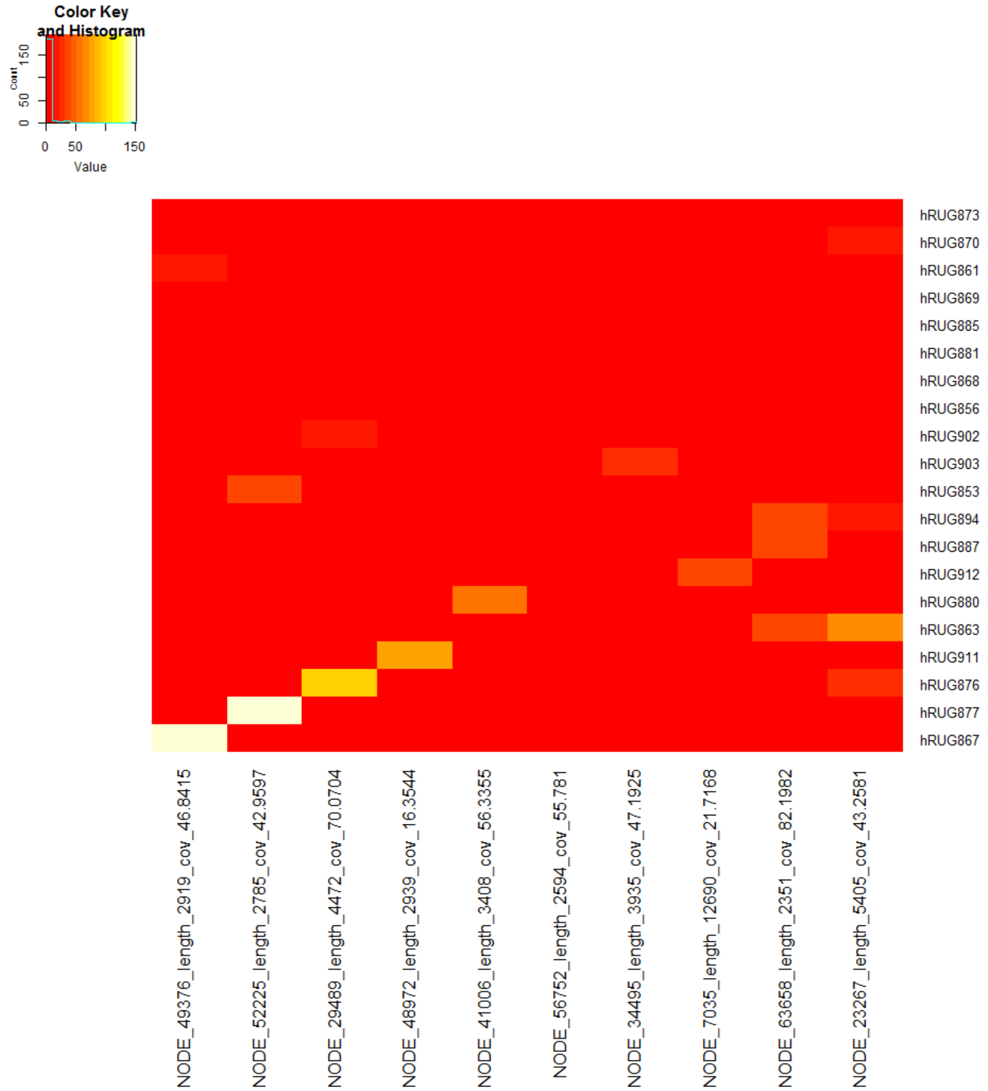

Supplementary Figure 5 Heatmap of the number of Hi-C links between 10 high coverage contigs with plasmid BLAST hits and 63 high quality hRUG genomes
