# Supplementary material for: Assembly of 913 microbial genomes from metagenomic sequencing of the cow rumen
Source: Nat Commun. 2018 Feb 28;9:870. doi: 10.1038/s41467-018-03317-6 (PMC5830445; doi:10.1038/s41467-018-03317-6)
Supplement: Supplementary file 2 — Description of Additional Supplementary Files [file 41467_2018_3317_MOESM2_ESM.pdf]

Description of Additional Supplementary Files

File Name: Supplementary Data 1
Description: Heatmap of abundance of RUGs across the 42 metagenomic samples. Abundance is calculated as the mean per-base read-coverage of each MAG within each sample. Values are log10 scaled.

File Name: Supplementary Data 2
Description: Description of the 913 Rumen Meta-Genomes (RUGs), including estimated taxon, CheckM completeness and contamination statistics, assembly size, N50, number of contigs and the longest contig length.

File Name: Supplementary Data 3
Description: Linear representation of Figure 1 comparing the RUGs with themselves and public genomes. Created using PhyloPhlAn and FigTree.

File Name: Supplementary Data 4
Description: Summary data from comparison of the RUGs against various databases using MinHash signatures.

File Name: Supplementary Data 5
Description: Summary data from a comparison of RUG proteomes against UniProt TrEMBL. Each RUG has total number of predicted proteins, the number with hits, the number predicted to be full length (qlen / hlen > 0.8), the most popular genus (and the number of proteins hitting that genus), the most popular organism (and the number of proteins hitting that organism) and the average percentage identity across all hits.

File Name: Supplementary Data 6
Description: Comparison of 28 archaeal RUGs with 597 publicly available archaeal genomes. Created using PhyloPhlAn and FigTree.

File Name: Supplementary Data 7
Description: Summary of the comparison of RUG proteomes against CAZy using dbCAN. Results show the relevant HMM hit, the length of the HMM, query ID and length, e-value, alignment details and the predicted coverage of the HMM in the protein.

File Name: Supplementary Data 8
Description: Summary data from a comparison of the RUG CAZy hits against various public databases. hmm_hit is the predicted CAZy HMM. Proteins were searched against nr, env_nr, m5nr, and Hess et al. data. Values are the maximum percentage identity recorded.

File Name: Supplementary Data 9
Description: Full results from a comparison of RUG proteomes against Pfam using pfam_can.

File Name: Supplementary Data 10
Description: Heatmap showing distribution of predicted CAZy enzymes across the 913 RUGs.

File Name: Supplementary Data 11
Description: Summary of the 1743 PULs.

File Name: Supplementary Data 12
Description: Full list of PULs including contig and protein names and predicted enzymes.

File Name: Supplementary Data 13
Description: Most common enzymes associated with PULs.

File Name: Supplementary Data 14
Description: 31 predicted Erysipelotrichales genomes placed into the tree of microbial life using PhyloPhlAn.

File Name: Supplementary Data 15
Description: A zoomed-in version of Supplementary Figure 6 showing only the Erysipelotrichales.

File Name: Supplementary Data 16
Description: Details of the 31 members of the Erysipelotrichales order.

File Name: Supplementary Data 17
Description: CheckM results for the 76 genome bins from the Hi-C metaSPAdes assembly created using MetaBAT2.

File Name: Supplementary Data 18
Description: BLAST results from contigs showing greater than twice the coverage of the average of their genome bin, searched against nt, filtered for “plasmid” in the title, and with short HSPs filtered out. All results are from the Hi-C genomes.

File Name: Supplementary Data 19
Description: The 850 RUG metagenome-assembled genomes ordered by coverage from low to high.
