# Supplementary material for: Assembly of 913 microbial genomes from metagenomic sequencing of the cow rumen
Source: Nat Commun. 2018 Feb 28;9:870. doi: 10.1038/s41467-018-03317-6 (PMC5830445; doi:10.1038/s41467-018-03317-6)

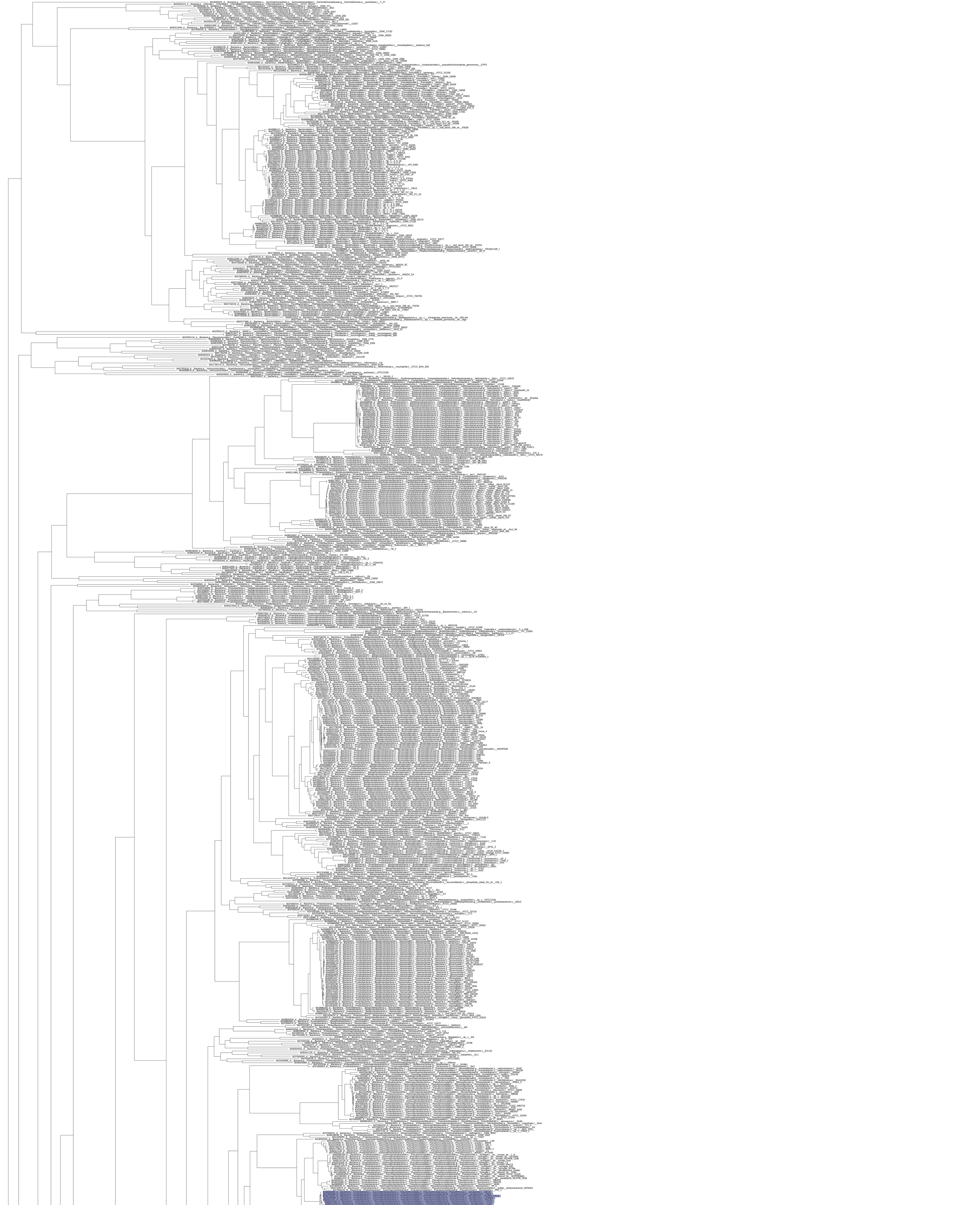

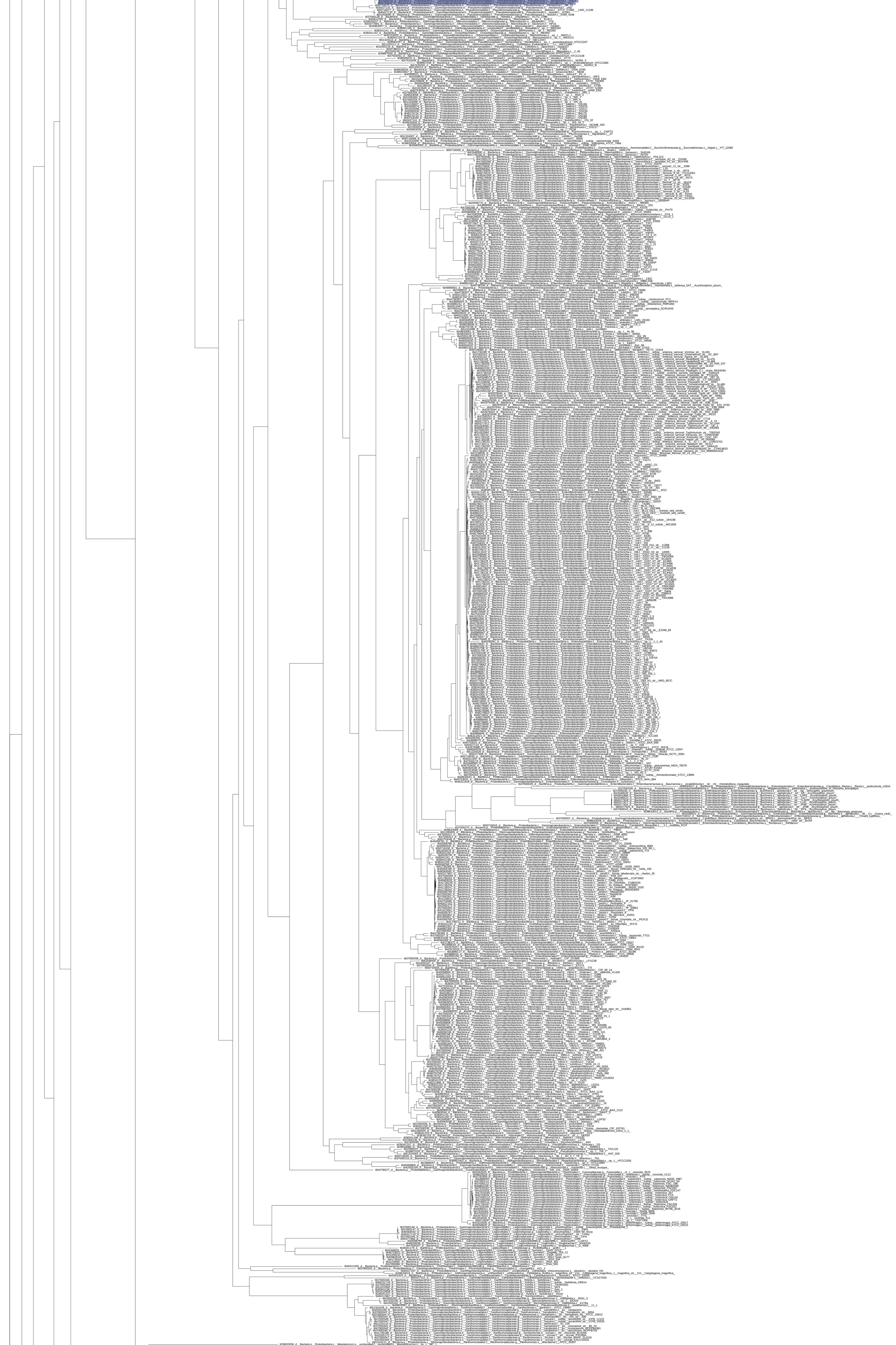

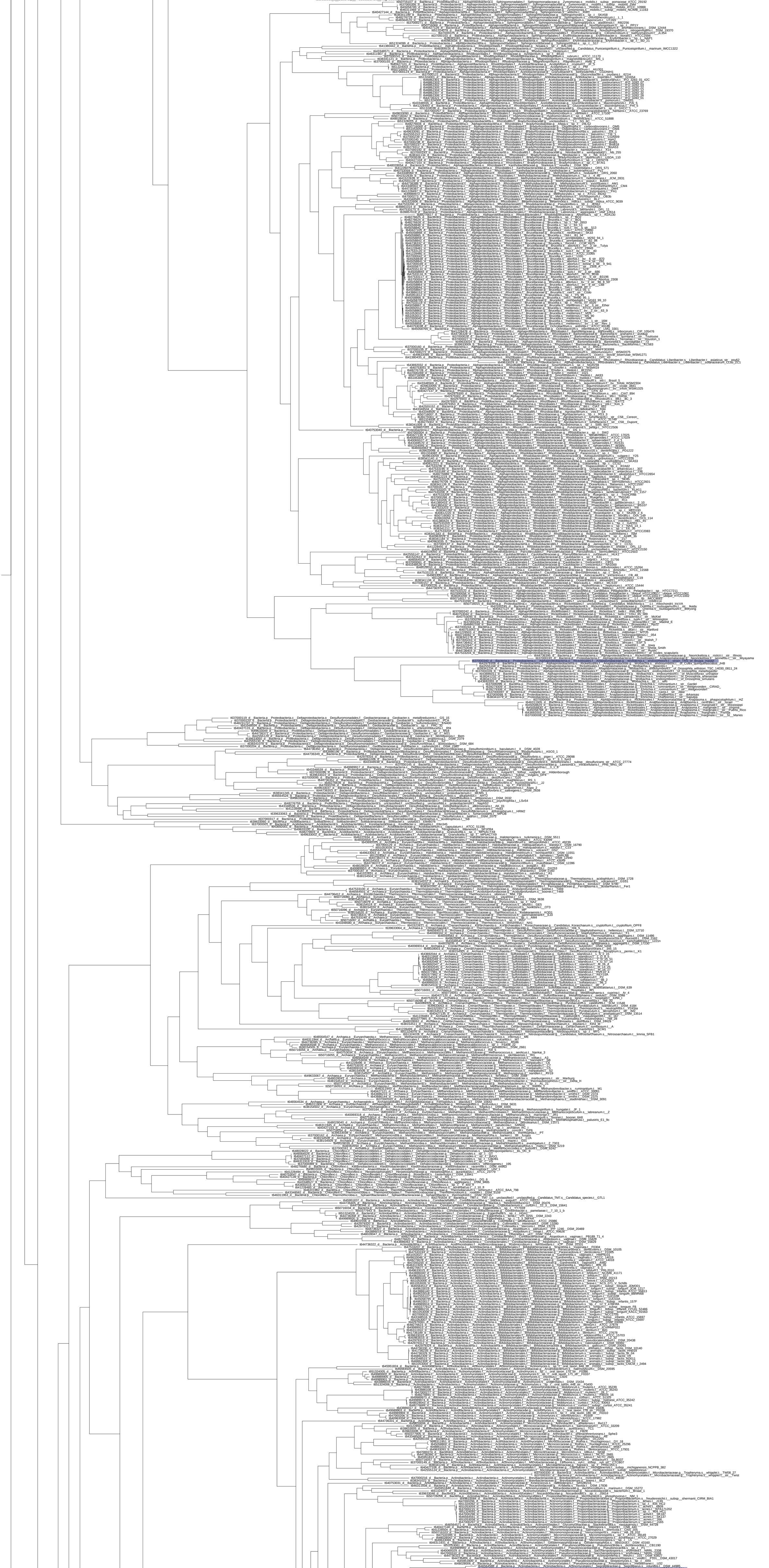

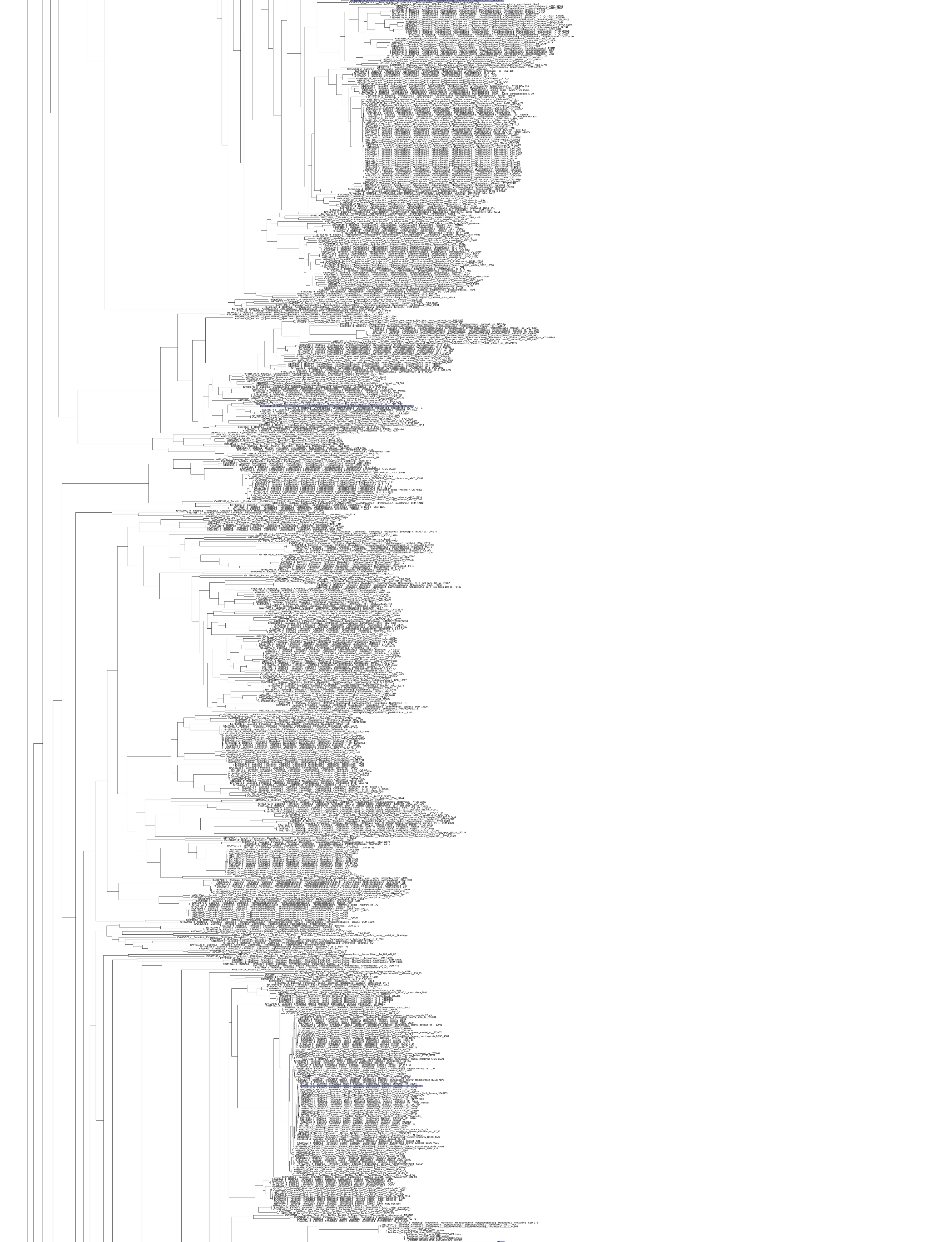

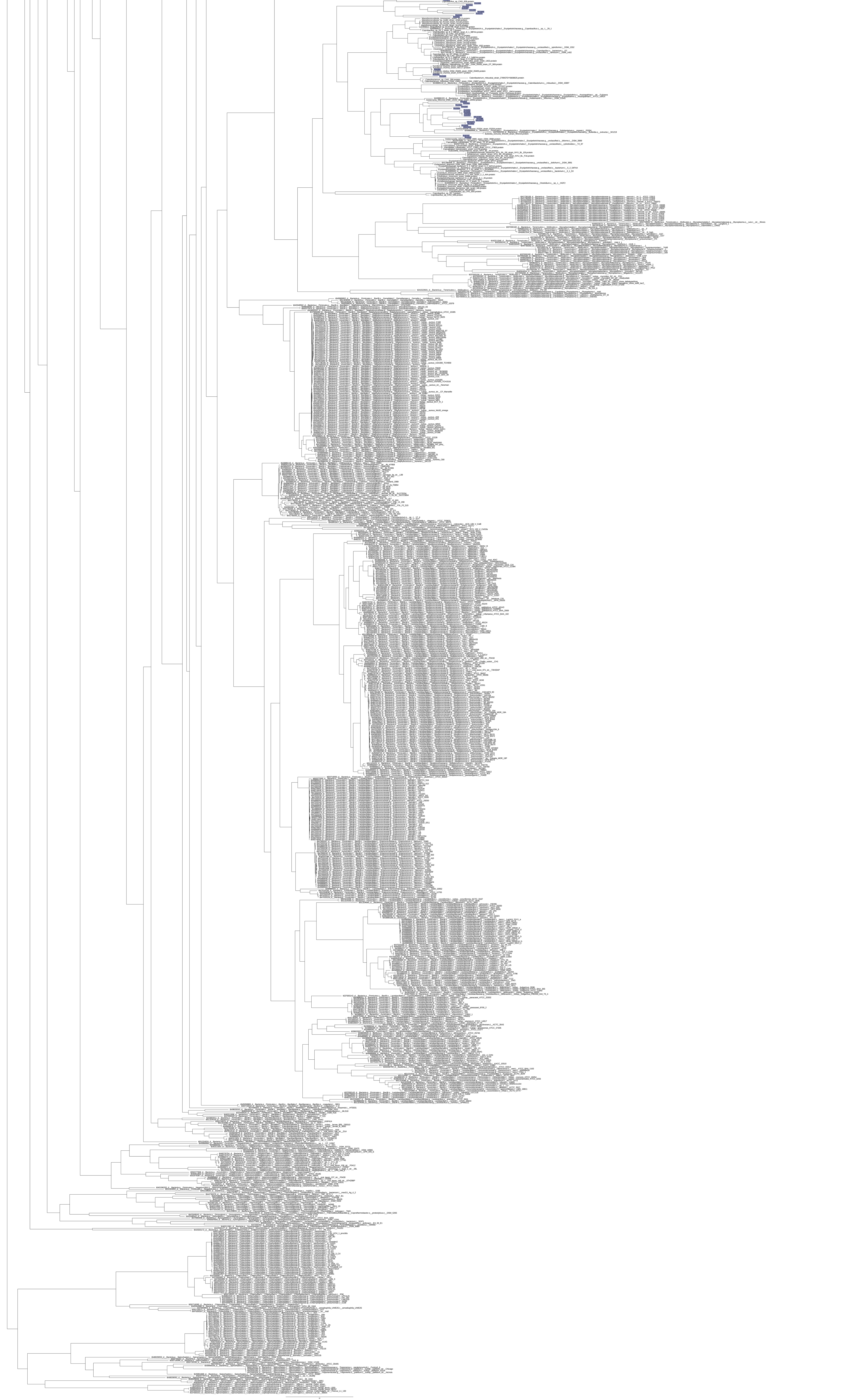

Supplement: Supplementary file 16 — Supplementary Data 14 [file 41467_2018_3317_MOESM16_ESM.pdf]
